# Dental caries thresholds among adolescents in England, Wales, and Northern Ireland, 2013 at 12, and 15 years: implications for epidemiology and clinical care

**DOI:** 10.1186/s12903-021-01507-1

**Published:** 2021-03-19

**Authors:** Xiaozhe Wang, Eduardo Bernabe, Nigel Pitts, Shuguo Zheng, Jennifer E. Gallagher

**Affiliations:** 1 Department of Preventive Dentistry, Peking University School and Hospital of Stomatology & National Clinical Research Center for Oral Diseases & National Engineering Laboratory for Digital and Material Technology of Stomatology & Beijing Key Laboratory of Digital Stomatology, 22 Zhongguancun South Avenue, Haidian District, Beijing, 100081 PR China; 2 Centre for Host Microbiome Interactions, King’s College London, Denmark Hill Campus, Bessemer Road, London, SE5 9RS UK; 3 Centre for Clinical and Translational Research, King’s College London, Guy’s Hospital Campus, Great Maze Pond, London, SE1 9RT UK; 4 Faculty of Dentistry, Oral and Craniofacial Sciences, King’s College London, Bessemer Road, London, SE5 9RS UK

**Keywords:** CDHS 2013, Dental caries, Distribution, Threshold, Risk factors

## Abstract

**Background:**

Dental caries is the most prevalent condition globally. Despite improvements over the past few decades, there remains a significant disease burden in childhood. Epidemiological surveys provide insight to disease patterns and trends, and have traditionally focused on *obvious decay* which are inconsistent with contemporary clinical criteria. This study examined the distribution of dental caries in 12- and 15-year-olds in England, Wales and Northern Ireland, by severity threshold, at surface, tooth and child level and explored its association with socioeconomic, psychological and behavioural factors.

**Methods:**

Data from 12- and 15-year-olds in the 2013 Children’s Dental Health Survey (CDHS 2013) were analysed at three levels, taking account of dental caries thresholds which involved recording both *clinical decay* [visual enamel caries (AV) and above] and *obvious decay* [non-cavitated dentine lesions (2V) and above]. Negative binomial regression was used to identify factors associated with dental caries experience at both thresholds.

**Results:**

The prevalence and severity of dental caries experience was higher among 15-year-olds at all levels. Visual change in enamel (AV) was by far the most common *stage* of caries recorded in both ages. The average number of surfaces with *obvious decay* experience, which has been the traditional epidemiological threshold, in 12- and 15-year-olds was 2.3 and 3.9 respectively. The corresponding values under the *clinical decay* threshold were higher, at 3.9 and 5.9 respectively. Visualisation of the distribution of dental caries at surface/tooth-level exhibited horizontal symmetry and to a lesser extent vertical symetry. In the adjusted models for both ages, country/region, school type, area deprivation, high frequency sugar consumption and irregular dental attendance were associated with greater caries experience in both groups. Dental anxiety was inversely associated with caries experience among 15-year-olds.

**Conclusion:**

This research highlights the importance of recognising dental caries patterns by surface, tooth and child-level amongst adolescents and the value of reporting dental caries distribution by threshold in epidemiological surveys, including its relevance for clinical care. Inclusion of enamel caries reveals the extent of caries management required at a point when non-invasive care is possible, emphasising the importance of prevention through contemporary primary care, which includes supporting self-care.

**Supplementary Information:**

The online version contains supplementary material available at 10.1186/s12903-021-01507-1.

## Background

Dental caries is the most common disease in the oral cavity globally [1]. A decline in caries experience measured by the decayed, missing and filled (DMFT/S) index has occurred in high-income countries over the past five decades [2–5]. Remarkably, neither the prevalence nor incidence of untreated caries in permanent teeth has changed between 1990 and 2015 in all ages globally [1, 6, 7].

Several studies have reported that enamel or non-cavitated caries lesions contribute much of the total caries prevalence in different populations and may be an indicator to help assess the risk status of an individual [8–10]. Recognising the process of dental caries development, and progression, clinical indices such as International Caries Detection and Assessment System (ICDAS) [11–14], helpfully map the profile of disease at individual level, recognising the importance of shifting from a restorative-approach towards early non-invasive management of disease [15, 16]. However, the total picture of the caries distribution including all stages of lesions among school-aged children in the United Kingdom (UK) has not been examined before.

The 2013 Children’s Dental Health Survey (CDHS 2013), commissioned by the Health and Social Care Information Centre, is the fifth in a series of national surveys of children’s oral health within the UK. Informed by ICDAS, enamel caries was recorded both at visual (non-cavitated) and cavitated level for the first time in this series of national surveys in 2013. This revealed that whilst just 34% of 12-year-olds and 46% 15-year-olds had *obvious decay* experience (non-cavitated lesions into dentine and above), 57% and 63% of them respectively had *clinical decay* experience when early caries (non-cavitated enamel caries and above) was included [17, 18]. Understanding the pattern of disease is fundamental to good contemporary dental caries management which involves early identification and managing the risk of further disease [1, 19].

The aims of this study were to examine the distribution of dental caries across a range of lesion-severity thresholds in 12- and 15-year-old children in England, Wales and Northern Ireland, at surface, tooth and individual levels, and explore its association with socioeconomic, psychological and behavioural factors.

## Methods

### Study population and data collection

This study involved secondary analysis of cross-sectional data from the CDHS 2013, collected in line with the published methodology [20, 21]. Three of the four nations of the UK, England, Wales, and Northern Ireland participated in this survey. A representative sample of eligible 12- (n = 2,532) and 15-year-olds (n = 2,418) from secondary schools in England, Wales and Northern Ireland was examined [17]. Pupils in two countries (Wales, Northern Ireland) and in more deprived schools were oversampled to facilitate reporting by country and relative deprivation [20]. Pupils were invited to complete a questionnaire survey at the same appointment exploring a range of issues including their self-rated health, oral symptoms and problems, impact of dental health on the quality of life, behavioural habits and psychological status [20], and a high response of 99.6% was achieved [17].

Dental examinations were undertaken in school settings by 75 trained and calibrated dentists. Training included pre-learning with a dedicated e-Learning programme and intensive lecture and in vivo sessions conducted in both classroom and school survey settings. Examiner calibration was conducted in 8 groups, and measured by calculating kappa scores, resulting in moderate to very good agreement on dental caries diagnosis [20]. Consent for the dental survey involved a decision to ‘opt-in’ by children on the day, with the possibility for parental ‘opt-out’ in advance. Visual examination was carried out using a plane mouth mirror and ball ended CPITN probe (WHO ball-ended probes) after drying with cotton wool/gauze; no radiographs were used [20]. Dental caries is a progressive and conceptually staged disease. In support of early detection of carious lesions, visual change in enamel was recorded as caries for the first time in the 2013 CDHS survey. The criteria used were consistent with the ICDAS [13, 14, 17]. Caries assessment was undertaken by surface and coded as follows: *sound* (including any sub-clinical decay); *visual change in enamel* (CDHS AV): ICDAS 1 and 2; *visual enamel change with cavitation* (CDHS AC): ICDAS 3; *visual dentine caries* (non-cavitated, CDHS 2V): ICDAS 4; *cavitated dentine caries* (CDHS 2C); and *decay with pulpal involvement* (CDHS 3): ICDAS 5 and 6. In addition, filled with recurrent decay (with/without cavitation), filling needs replacement, sound fillings (F), and extracted due to caries (M) were also recorded [17, 20]. Dental caries activity was not assessed. Two visual detection thresholds were used to assess caries status in this research, which were presented as *clinical decay* (CDHS AV and above), and *obvious decay* (CDHS 2V and above). Decay experience in terms of these criterion includes currently and previously decayed teeth/surfaces, and were reported as D_AV_MFT/S, D_2V_MFT/S respectively. All methods in the study were carried out in accordance with the Helsinki guidelines and declaration.

### Data management

Key sociodemographic factors were included in this analysis as potential confounders [7, 22]. Sex (male/female) and ethnicity (white/non-white) were treated as dichotomous variables. Government Office Region was a 11-category variable, constituted by nine regions in England, plus the two countries of Wales, and North Ireland. School type was grouped into three categories: independent, secondary and academy or free school. There were two indicators for family and contextual socioeconomic status used within the analysis. One was free school meal eligibility, a statutory benefit only provided to disadvantaged pupils in maintained schools, academies, and free schools. The other was the IMD score (Index of Multiple Deprivation) based on indicators of distinct dimensions of deprivation categories, by quintile, for each country on the basis of the overall score [23–25].

Toothbrushing frequency was reported as a binary categorical variable (twice a day or more versus once a day or less). Consideration of frequency of sugar intake—involved aggregating reported daily consumption of several common sugary foods and drinks (sweets, biscuits, cakes, fruits, soft drinks that contain sugar, energy/sports drinks, and fruit juice or smoothies) into a total score, which was recategorised into a binary variable (less than four times a day or four or more times a day). Because only 2.41% of participants reported having “never been to the dentist”, the reason for usual dental attendance was dichotomized into regular (for a check-up) versus all the rest which is irregular (only when have trouble)/none. The Modified Dental Anxiety Scale (MDAS) was first introduced to CDHS in 2013. It consists of 5 questions, each with a 5-category rating scale, ranging from ‘not anxious’ to ‘extremely anxious’. The self-rated dental anxiety score was grouped into three categories for analysis (5–9 indicating low/no anxiety, 10–18 representing moderate anxiety, and 19–25 as extreme anxiety).

### Statistical analysis

First, we calculated the distribution and composition of different stages or thresholds of dental caries (*clinical* and *obvious decay)* across the permanent dentition at surface, tooth, and child level for 12- and 15-years-olds. Second, the characteristics of participants according to relevant socio-demographic factors, behavioural and psychological factors were examined. The distribution of excluded pupils was compared with research samples in relevant variables by using Chi-square test to evaluate the impact of missing data. Complex survey design (stratification and clustering) was taken into consideration by using Negative binomial regression [20]. Third, to test the association between dental behavioural, psychological factors and caries experience (DMFS_AV/2V_ index), an unadjusted model and an adjusted model were successively built using negative binomial regression. The association of toothbrushing frequency, frequency of sugar intake, usual dental attendance, and dental anxiety with dental caries experience (D_AV/2V_MFS) were estimated. Potential confounders including demographic status were introduced into the model to make an adjusted estimation. Rate ratios (RRs), 95% confidence intervals (95% CI) and level of significance were reported and compared in all models. All analyses were conducted using Stata/SE 15 (StataCorp LLC, College Station, TX). *P* < 0.05 was considered as statistically significant.

## Results

The distribution of dental caries in the permanent dentition was presented at surface, tooth, and child-level, by caries stage/threshold, for both ages examined in the national survey (Tables 1, 2). At all three levels, the prevalence and average number of surfaces or teeth with dental caries experience was higher in 15-year-olds compared with 12-year-old children.

**Table 1:** Dental caries distribution at surface, tooth and child levels amongst 12-year-olds^a,b^

Distribution according to CDHS 2013 code	Surface level				Tooth level^c^				Child level^c^	
	Sum	%	Mean/child	S.D	Sum	%	Mean/child	S.D	Sum	%
Sound ^d^	288,502	96.68	113.94	16.15	59,285	90.38	23.41	4.20	890	35.15
Code AV-visual change in enamel	3718	1.25	1.47	2.79	2841	4.33	1.12	2.02	431	17.02
Code AC-enamel change with cavitation	359	0.12	0.14	0.58	314	0.48	0.12	0.50	75	2.96
Code 2V-visual dentine caries	1157	0.39	0.46	1.42	819	1.25	0.32	0.90	174	6.87
Code 2C-cavitated dentin caries	743	0.25	0.29	1.14	515	0.79	0.20	0.70	221	8.73
Code 3-decay with pulpal involvement	414	0.14	0.16	1.32	108	0.16	0.04	0.31	57	2.25
Filled with recurrent decay	191	0.06	0.08	0.46	144	0.22	0.06	0.33	80	3.16
Filling needs replacement, no decay	25	0.01	0.01	0.17	14	0.02	0.01	0.08	5	0.20
Sound filling	2001	0.67	0.79	1.83	1290	1.97	0.51	1.06	470	18.56
Missing due to decay	1310	0.44	0.52	2.60	262	0.40	0.10	0.52	129	5.09
Total	298,420	100.00			65,592	100.00			2532	100.00

^a^Counts and proportions are unweighted

^b^2013 Children’s Dental Health Survey included England, Wales, and Northern Ireland

^c^Codes at tooth and child level represent the worst code in each tooth and child

^d^Including sub-clinical decay and lesions seen only on radiographs

**Table 2:** Dental caries distribution at surface, tooth and child levels amongst 15-year-olds^a,b^

Distribution according to CDHS 2013 code	Surface level				Tooth level^c^				Child level^c^	
	Sum	%	Mean/child	S.D	Sum	%	Mean/child	S.D	Sum	%
Sound^d^	292,155	95.06	120.83	9.14	58,104	86.44	24.03	4.19	643	26.59
Code AV-visual change in enamel	4356	1.42	1.80	3.32	3266	4.86	1.35	2.35	289	11.95
Code AC-enamel change with cavitation	528	0.17	0.22	0.76	429	0.64	0.18	0.63	60	2.48
Code 2V-visual dentine caries	1319	0.43	0.55	1.50	959	1.43	0.40	1.03	237	5.05
Code 2C-cavitated dentin caries	764	0.25	0.32	1.20	541	0.80	0.22	0.74	192	7.94
Code 3-decay with pulpal involvement	395	0.13	0.16	1.47	105	0.16	0.04	0.35	28	1.16
Filled with recurrent decay	310	0.10	0.13	1.03	219	0.33	0.09	0.52	101	4.18
Filling needs replacement, no decay	61	0.02	0.03	0.24	34	0.05	0.01	0.13	10	0.58
Sound filling	5038	1.64	2.08	3.80	3081	4.58	1.27	2.03	608	29.74
Missing due to decay	2415	0.79	1.00	3.50	483	0.72	0.20	0.70	250	10.34
Total	307,341	100.00			67,221	100.00			2418	100.00

^a^Counts and proportions are unweighted

^b^2013 Children’s Dental Health Survey included England, Wales, and Northern Ireland

^c^Codes at tooth and child level represent the worst code in each tooth and child

^d^Including sub-clinical decay and lesions seen only on radiographs

When considering *obvious decay*, the average number of decayed surfaces in 12- and 15-year-olds was only 0.99 and 1.16, respectively. The volume of recorded disease was higher when using the *clinical decay* threshold (which incorporated enamel caries) up to 2.74 times higher in 12- and 15-year-olds at 2.60 and 3.18, respectively.

The findings revealed that 3.32% of surfaces and 9.62% teeth in 12-year-olds, and 4.94% surfaces and 13.56% teeth in 15-year-olds had *clinical decay* experience (D_AV_MFS/T). 41% of the surfaces and 50% of teeth with decay-experience were enamel caries in 12-year-olds, while these proportions were lower in 15-year-olds at 32% and 41% respectively. Past decay experience, managed through extractions and fillings (missing or filled teeth), occupied a larger portion of decay experience in both age groups. It is important, therefore, to recognise the dramatic finding that by far the most frequent “stage” of lesions encountered in 12- and 15-year-olds was visual enamel caries (AV).

**Fig. 1:**
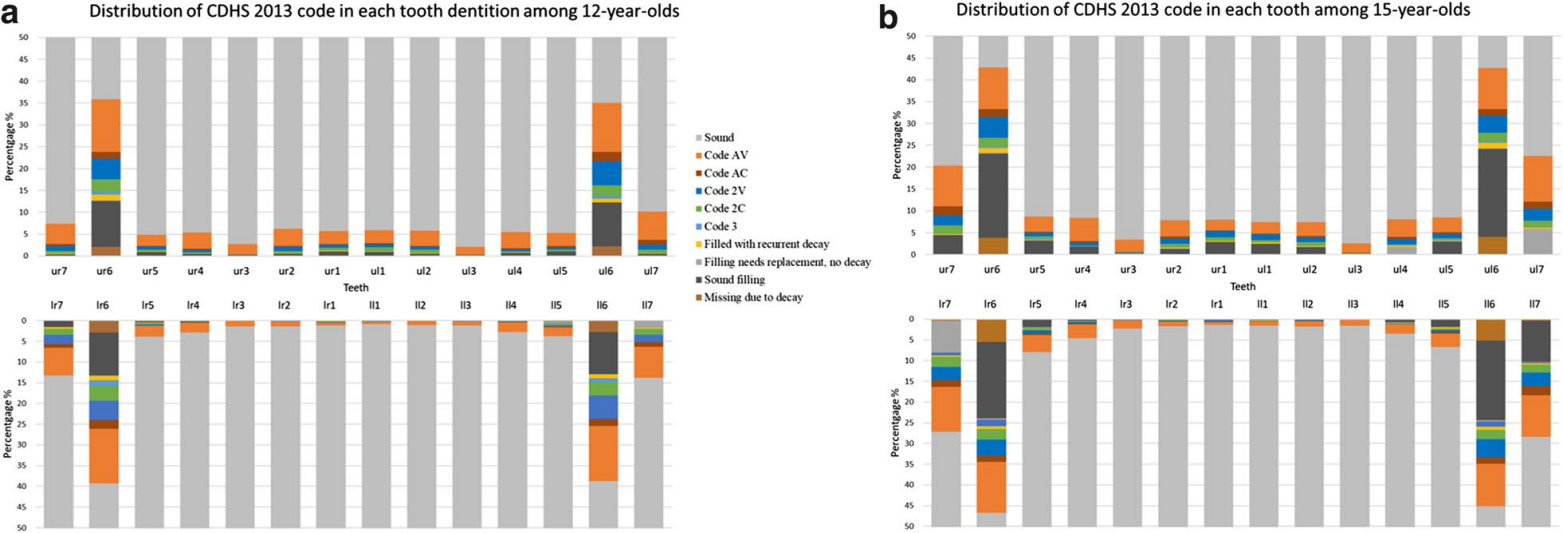
Distribution of dental caries by tooth amongst 12- and 15-year-olds^a^. ^a^A representative sample of eligible 12- (n = 2532) and 15-year-olds (n = 2418) from 2013 Children’s Dental Health Survey in England, Wales and Northern Ireland was involved

Dental decay prevalence by stage is presented visually by at tooth and surface in Fig. 1 and Additional file 1: Appendix 1 respectively. Occlusal surfaces of all molars, buccal surfaces of lower first molars, and then smooth surfaces of upper first molars and buccal surfaces of lower second molars were most likely to be attacked by dental caries. The level of dental caries experience was higher in 15-year-olds, most notably in first and second molars. Caries prevalence amongst lower anterior and upper canine teeth remained low.

Overall, there was generally horizontal (right/left) symmetry, and to a lesser extent, vertical (upper/lower) symmetry. First molar teeth, which had been longest in the oral cavity exhibited comparable *clinical* decay prevalence of 35–39% across the four quadrants in 12-year-olds, rising to between 42 and 46% in 15-year-olds. There was little difference observed between upper/lower or left/right dentitions, merely more teeth extracted due to decay in 15-year-olds. Second molars in same dentition on both sides of the dental arch shared similar decay status with the prevalence of *clinical decay* experience whilst maxillary decay was lower and milder than mandibular in both age groups. Amongst pupils aged 12 years who had decayed lower second molars, only 47% of them had decayed upper second molars, this proportion increasing to 68% in 15-year-olds (Fig. 1).

**Fig. 2:**
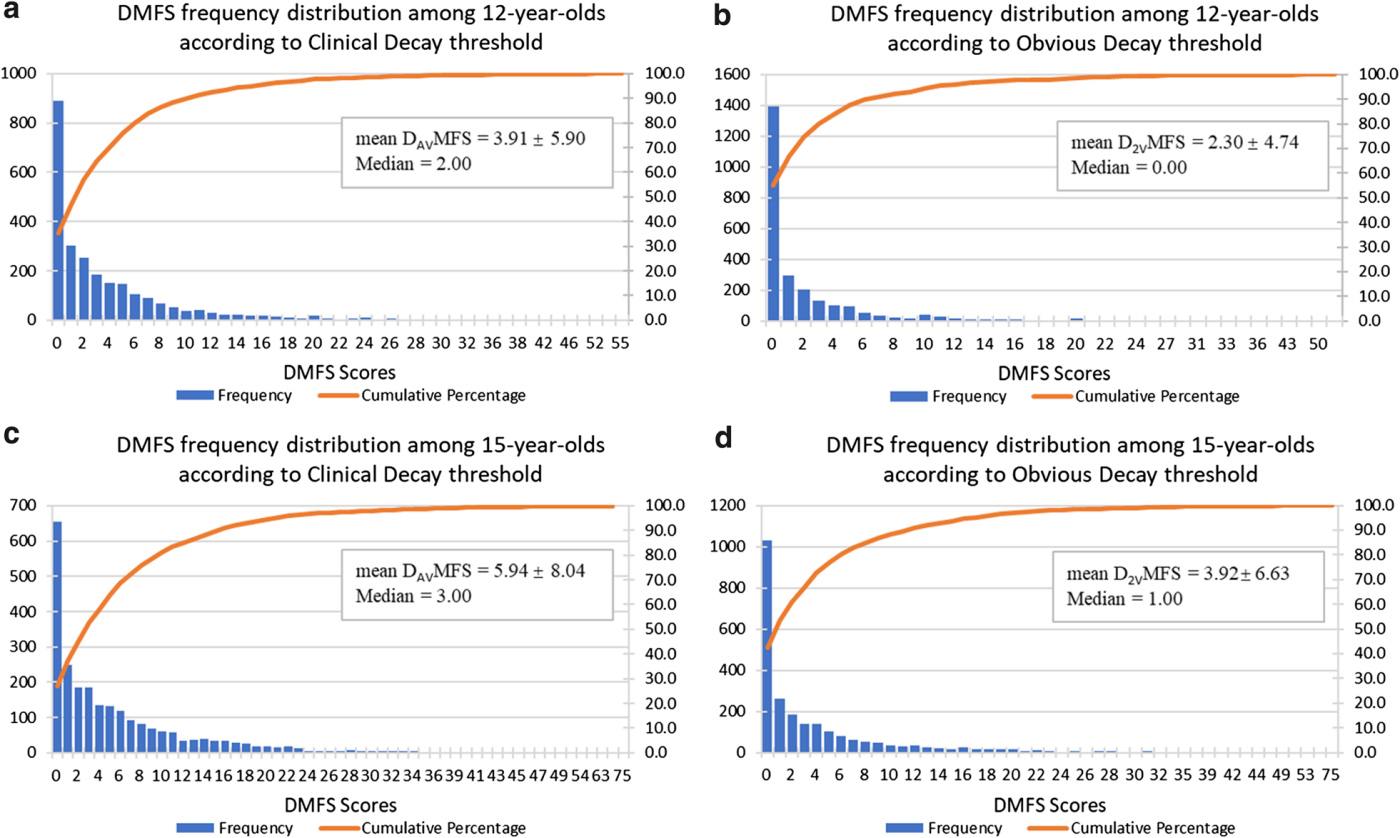
Frequency distribution of DMFS score by *clinical* and *obvious decay* threshold of 12- and 15-year-olds^a,b^. ^a^*Clinical decay* threshold (D_AV_MFS) represents CDHS AV and above. *Obvious decay* threshold (D_2V_MFS) represents CDHS 2 V and above. ^b^Counts, proportions and means are unweighted

The distribution of *clinical* and *obvious decay* exhibited marked differences amongst the 12- and 15-year-old populations as shown in Fig. 2. The average number of surfaces suffered from *obvious decay* experience was 2.3 in 12-year-olds and 3.9 in 15-year-olds, then this rose to 3.9 in 12-year-olds and 5.9 in 15-year-olds, when the *clinical decay* threshold including enamel caries was considered.

The multivariate analysis involved 1964 12-year-old and 1963 15-year-old pupils with complete data for all relevant variables to further explore associated factors with *clinical* and *obvious decay* experience. The characteristics of children at both ages are presented in Table 3. Behavioural and psychological factors involved in this analysis, including toothbrushing frequency, sugar intake frequency, dental attendance and dental anxiety were significantly associated with *clinical* and *obvious decay* experience in the unadjusted models at one or both ages (Tables 4, 5).

**Table 3:** Characteristics of 12- and 15-year-olds according to *Clinical* and *Obvious Decay* thresholds^a^

Variables	12-year-olds							15-year-olds						
	N	Weighted % [95% CI]		Mean D_AV_MFS		Mean D_2V_MF		N	Weighted % [95% CI]		Mean D_AV_MFS		Mean D_2V_MFS	
				*Clinical decay* [95% CI]		*Obvious decay* [95% CI]					*Clinical decay* [95% CI]		*Obvious decay* [95% CI]	
*Sex*														
Male	955	50.97	[44.78, 57.12]	2.74	[2.13, 3.36]	1.32	[0.96, 1.67]	936	47.94	[41.25, 54.71]	4.17	[3.33, 5.01]	2.52	[1.95, 3.08]
Female	1009	49.03	[42.88, 55.22]	2.99	[2.23, 3.76]	1.66	[1.10, 2.21]	1027	52.06	[45.29, 58.75]	4.40	[3.46, 5.33]	2.74	[2.17, 3.31]
*Free school meal eligibility*														
Not eligible for free school meals	1432	79.02	[74.93, 82.61]	2.41	[1.95, 2.86]	1.11	[0.89, 1.32]	1518	83.75	[79.01, 87.60]	3.96	[3.15, 4.78]	2.34	[1.8, 2.78]
Eligible for free school meals	532	20.98	[17.39, 25.07]	4.60	[3.40, 5.80]	2.90	[1.95, 3.85]	445	16.25	[12.40, 20.99]	5.96	[4.67, 7.24]	4.16	[3.35, 4.97]
*Region*														
London	138	10.84	[7.03, 16.35]	2.00	[1.12, 2.87]	1.31	[0.48, 2.15]	121	9.41	[6.79, 12.91]	3.40	[2.29, 4.52]	2.53	1.68, 3.39]
South East	122	16.55	[8.77, 29.04]	1.33	[0.33, 2.33]	0.55	[0.25, 0.85]	108	16.68	[7.69, 32.49]	2.58	[1.44, 3.72]	1.78	[1.05, 2.52]
East of England	123	11.47	[6.31, 19.97]	2.97	[1.76, 4.18]	1.64	[0.73, 2.55]	105	11.90	[5.63, 23.43]	2.61	[0.36, 4.87]	1.22	[0.15, 2.30]
West Midlands	118	11.45	[7.03, 18.09]	1.71	[1.32, 2.11]	0.98	[0.69, 1.27]	125	11.97	[9.00, 15.76]	3.41	[2.42, 4.39]	2.68	[1.54, 3.83]
East Midlands	79	8.24	[1.74, 31.34]	3.72	[3.41, 4.03]	1.47	[0.94, 1.99]	82	7.39	[1.77, 26.11]	4.91	[3.04, 6.78]	2.54	[1.12, 3.96]
Yorkshire and the Humber	125	9.36	[5.06, 16.69]	2.69	[2.01, 3.37]	0.91	[0.35, 1.46]	115	10.44	[5.43, 19.13]	4.60	[1.67, 7.52]	2.17	[1.06, 3.29]
North East	138	4.65	[2.13, 9.87]	3.42	[2.27, 4.58]	2.15	[1.47, 2.82]	158	5.10	[2.40, 10.51]	5.43	[3.21, 7.64]	4.07	[2.43, 5.70]
South West	96	7.22	[3.80, 13.3]	1.79	[0.31, 3.27]	0.97	[0.34, 1.61]	83	7.48	[4.19, 13.02]	4.02	[0.64, 7.39]	2.27	[0.69, 3.85]
Wales	482	5.23	[3.37, 8.33]	4.35	[3.74, 4.97]	2.34	[1.73, 2.96]	439	5.75	[3.58, 9.12]	6.18	[4.75, 7.62]	4.01	[2.90, 5.11]
North West	170	11.42	[4.64, 25.47]	5.82	[4.58, 7.05]	3.04	[1.66, 4.43]	188	10.19	[4.87, 20.10]	7.32	[5.28, 9.37]	3.61	[2.33, 4.88]
Northern Ireland	373	3.46	[2.47, 4.83]	4.32	[3.29, 5.36]	2.93	[2.37, 3.48]	439	3.67	[2.72, 4.92]	8.11	[6.41, 9.82]	6.52	[5.25, 7.79]
*School type*														
Independent school	101	6.52	[2.40, 16.49]	1.16	[0.24, 2.08]	0.37	[0.16, 0.58]	81	8.22	[2.29, 25.49]	1.52	[0.29, 2.75]	0.76	[0.57, 0.95]
Academy or free school	429	37.74	[24.75, 52.77]	2.70	[1.97, 3.43]	1.32	[0.93, 1.71]	410	37.33	[24.85, 51.76]	3.71	[2.49, 4.93]	2.22	[1.45, 2.99]
Secondary school	1434	55.74	[39.82, 70.56]	3.18	[2.52, 3.84]	1.73	[1.26, 2.19]	1472	54.46	[37.82, 70.15]	5.11	[4.23, 5.99]	3.20	[2.68, 3.72]
*Index of multiple deprivation quintile*														
80%-100% Least deprived	212	15.28	[2.41, 23.71]	1.47	[0.91, 2.02]	0.56	[0.32, 0.79]	210	15.10	[10.00, 22.15]	3.07	[1.74, 4.41]	1.60	[0.80, 2.39]
60%-80%	297	19.06	[14.65, 24.42]	1.92	[1.24, 2.61]	0.73	[0.43, 1.03]	290	18.83	[13.23, 26.08]	3.89	[1.99, 5.80]9	2.03	[1.06, 3.01]
40%-60%	317	12.56	[8.84, 17.54]	3.13	[2.20, 4.06]	1.79	[1.11, 2.46]	311	16.17	[12.83, 20.19]	3.69	[2.55, 4.83]	2.29	[1.44, 3.13]
20%-40%	406	19.28	[15.3, 23.98]	2.74	[2.12, 3.35]	1.54	[0.96, 2.12]	428	19.51	[15.20, 24.69]	4.41	[3.60, 5.23]	3.00	[2.44, 3.55]
0–20% Most deprived	732	33.82	[25.43, 43.38]	4.01	[3.01, 5.01]	2.18	[1.40, 2.96]	724	30.39	[21.76, 40.67]	5.38	[4.16, 6.60]	3.47	[2.39, 4.54]
*Ethnicity*														
White	1647	80.83	[74.30, 86.01]	3.08	[2.52, 3.64]	1.62	[1.23, 2.00]	1639	81.17	[75.46, 85.80]	4.35	[3.36, 5.34]	2.63	[2.06, 3.19]
Non-white	317	19.17	[13.99, 25.7]	1.97	[1.40, 2.53]	0.93	[0.56, 1.30]	324	18.83	[14.20, 24.54]	4.03	[3.15, 4.92]	2.65	[1.77, 3.52]
*Frequency of brushing teeth*														
Twice a day or more	1499	77.61	[73.59, 81.17]	2.57	[2.07, 3.08]	1.38	[1.03, 1.73]	1545	80.81	[77.95, 83.37]	4.01	[3.25, 4.77]	2.45	[2.00, 2.89]
Once a day or less	465	22.39	[18.83, 26.41]	3.88	[2.66, 5.11]	1.86	[1.20, 2.51]	418	19.19	[16.63, 22.05]	5.46	[4.10, 6.81]	3.42	[2.32, 4.52]
*Frequency of sugar intake*														
Less than four times a day	650	35.02	[30.97, 39.39]	2.04	[1.56, 2.53]	1.03	[0.66, 1.41]	683	38.42	[34.64, 42.35]	3.17	[2.23, 4.11]	1.80	[1.34, 2.26]
Four or more times a day	1314	64.98	[60.71, 69.03]	3.31	[2.72, 3.90]	1.73	[1.31, 2.14]	1280	61.58	[57.65, 65.36]	4.99	[4.1, 5.80]	3.15	[2.60, 3.70]
*Usual dental attendance*^*b*^														
Regular	1677	84.55	[81.14, 87.44]	2.61	[2.17, 3.05]	1.29	[1.06, 1.52]	1653	84.86	[80.86, 88.15]	3.72	[2.94, 4.49]	2.13	[1.72, 2.54]
Irregular/none	287	15.45	[12.56, 18.86]	4.27	[2.71, 5.83]	2.52	[1.40, 3.64]	310	15.14	[11.85, 19.14]	7.50	[5.84, 9.16]	5.46	[4.22, 6.69]
*Self-rated dental anxiety score MDAS grouping*														
Low/no anxiety (score 5–9)	551	24.78	[21.8, 28.02]	3.52	[2.69, 4.36]	1.73	[1.24, 2.21]	708	36.21	[32.40, 40.20]	4.99	[3.98, 6.00]	3.12	[2.44, 3.81]
Moderate anxiety (score 10–18)	1163	62.19	[58.74, 65.51]	2.52	[1.99, 3.05]	1.31	[1.02, 1.60]	1014	54.01	[49.52, 58.42]	3.60	[2.76, 4.44]	2.07	[1.63, 2.52]
Extreme anxiety (score 19–25)	250	13.04	[11.02, 15.36]	3.26	[2.11, 4.41]	1.84	[0.99, 2.68]	241	9.78	[7.80, 12.20]	5.50	[4.15, 6.84]	3.91	[2.72, 5.10]
Total	1964	100		2.87	[2.39, 3.34]	1.48	[1.18, 1.78]	1963	100		4.29	[3.51, 5.07]	2.63	[2.18, 3.08]

^a^*Clinical decay* threshold (D_AV_MFS) represents CDHS AV and above. *Obvious decay* threshold (D_2V_MFS) represents CDHS 2 V and above

^b^Regular represents “for a check-up”, irregular/none combines “only when have trouble with teeth” and “never been to the dentist”

**Table 4:** Association of dental behaviours, diet and dental anxiety with D_AV_MFS and D_2V_MFS in 12-year-olds^a^

Variables	*Clinical decay* threshold				*Obvious decay* threshold			
	Unadjusted model^b^		Adjusted model^b^		Unadjusted model^b^		Adjusted model^b^	
	RR [95% CI]		RR [95% CI]		RR [95% CI]		RR [95% CI]	
*Sex*								
Male	1.00	[Reference]	1.00	[Reference]	1.00	[Reference]	1.00	[Reference]
Female	1.17	[1.02, 1.34]	1.13	[0.90, 1.42]	1.30	[1.08, 1.56]	1.16	[0.83, 1.62]
*Free school meal eligibility*								
Not eligible for free school meals	1.00	[Reference]	1.00	[Reference]	1.00	[Reference]	1.00	[Reference]
Eligible for free school meals	1.73	[1.49, 2.00]***	1.55	[1.38, 2.10]**	2.09	[1.72, 2.55]***	2.13	[1.52, 2.99]***
*Region*								
London	1.00	[Reference]	1.00	[Reference]	1.00	[Reference]	1.00	[Reference]
South East	1.01	[0.70, 1.46]	1.12	[0.51, 2.49]	0.61	[0.36, 1.02]*	1.17	[0.54, 2.56]
East of England	1.46	[1.02, 2.09]	2.04	[1.16, 3.55]**	1.22	[0.74, 1.99]	2.55	[1.16, 5.61]*
West Midlands	0.87	[0.60, 1.27]	1.26	[0.77, 2.07]	0.74	[0.44, 1.23]	1.32	[0.67, 2.62]
East Midlands	2.10	[1.41, 3.15]	1.65	[0.97, 2.79]	1.95	[1.13, 3.38]	1.05	[0.45, 2.49]
Yorkshire and the Humber	1.30	[0.91, 1.87]	2.37	[1.44, 3.90]***	1.02	[0.63, 1.68]	1.98	[0.75, 5.25]
North East	1.56	[1.10, 2.22]**	2.25	[1.32, 3.82]**	1.49	[0.93, 2.40]	2.73	[1.22, 6.08]*
South West	0.83	[0.56, 1.24]***	1.44	[0.63, 3.26]	0.74	[0.43, 1.28]	1.95	[0.84, 4.50]
Wales	1.98	[1.49, 2.63]***	2.85	[1.77, 4.57]***	1.81	[1.23, 2.65]	3.14	[1.59, 6.20]***
North West	3.11	[2.24, 4.32]**	3.09	[1.99, 4.80]***	2.51	[1.60, 3.92]*	3.02	[1.65, 5.51]***
Northern Ireland	2.59	[1.94, 3.46]	3.11	[1.78, 5.43]***	2.94	[1.99, 4.35]*	4.72	[2.25, 9.89]***
*School type*								
Independent school	1.00	[Reference]	1.00	[Reference]	1.00	[Reference]	1.00	[Reference]
Academy or free school	1.51	[1.07, 2.11]*	1.42	[0.73, 2.76]	2.87	[1.75, 4.72]***	1.87	[1.23, 2.85]**
Secondary school	2.11	[1.54, 2.90]*	1.39	[0.63, 3.07]	4.86	[3.04, 7.77]***	1.93	[1.21, 3.09]**
*Index of multiple deprivation quintile*								
80–100% Least deprived	1.00	[Reference]	1.00	[Reference]	1.00	[Reference]	1.00	[Reference]
60–80%	1.30	[0.99, 1.70]	1.21	[0.78, 1.87]	1.27	[0.88, 1.85]	1.22	[0.79, 1.90]
40–60%	1.46	[1.12, 1.90]**	2.00	[1.35, 2.98]**	1.93	[1.34, 2.77]***	3.05	[1.87, 4.98]***
20–40%	1.82	[1.41, 2.34]**	1.47	[0.97, 2.24]	2.28	[1.61, 3.22]***	2.28	[1.28, 4.06]**
0–20% Most deprived	2.26	[1.79, 2.86]***	2.29	[1.57, 3.33]***	2.96	[2.15, 4.09]***	3.83	[2.39, 6.12]***
*Ethnicity*								
White	1.00	[Reference]	1.00	[Reference]	1.00	[Reference]	1.00	[Reference]
Non-white	0.75	[0.62, 0.90]*	0.66	[0.50, 0.88]**	0.56	[0.43, 0.72]*	0.50	[0.33, 0.77]**
*Frequency of brushing teeth*								
Twice a day or more	1.00	[Reference]	1.00	[Reference]	1.00	[Reference]	1.00	[Reference]
Once a day or less	1.51	[1.03, 2.22]*	1.26	[0.95, 1.66]	1.35	[0.86, 2.11]	1.19	[0.87, 1.64]
*Frequency of sugar intake*								
Less than four times a day	1.00	[Reference]	1.00	[Reference]	1.00	[Reference]	1.00	[Reference]
Four or more times a day	1.62	[1.23, 2.13]***	1.38	[1.06, 1.78]*	1.67	[1.07, 2.62]*	1.32	[0.83, 2.09]
*Usual dental attendance *^*c*^								
Regular	1.00	[Reference]	1.00	[Reference]	1.00	[Reference]	1.00	[Reference]
Irregular/none	1.73	[1.44, 2.08]**	1.51	[1.11, 2.05]**	1.95	[1.52, 2.51]**	1.95	[1.32, 2.88]***
*Self-rated dental anxiety score MDAS grouping*								
Low/no anxiety (score 5–9)	1.00	[Reference]	1.00	[Reference]	1.00	[Reference]	1.00	[Reference]
Moderate anxiety (score 10–18)	0.78	[0.67, 0.91]*	0.81	[0.62, 1.05]	0.78	[0.64, 0.96]*	0.81	[0.56, 1.18]
Extreme anxiety (score 19–25)	1.16	[0.93, 1.45]	0.80	[0.61, 1.06]	1.43	[1.06, 1.93]	0.77	[0.47, 1.26]

^a^D_AV_MFS (CDHS AV and above) represents decay experience *according to clinical decay* threshold. D_2V_MFS (CDHS 2 V and above) represents decay experience *obvious decay* threshold

^b^Unadjusted and full-adjusted Negative binomial regression models were fitted, rate ratios (RR) were reported

^c^Regular represents “for a check-up”, irregular/none combines “only when have trouble with teeth” and “never been to the dentist”

*p < 0.05, **p < 0.01, ***p < 0.001

**Table 5:** Association of dental behaviours, diet and dental anxiety with D_AV_MFS and D_2V_MFS in 15-year-olds^a^

Variables	Clinical decay threshold				Obvious decay threshold			
	Unadjusted model^b^		Adjusted model^b^		Unadjusted model^b^		Adjusted model^b^	
	RR [95% CI]		RR [95% CI]		RR [95% CI]		RR [95% CI]	
*Sex*								
Male	1.00	[Reference]	1.00	[Reference]	1.00	[Reference]	1.00	[Reference]
Female	1.19	[1.05, 1.35]	1.24	[1.02, 1.50]*	1.36	[1.17, 1.58]	1.32	[1.01, 1.72]*
*Free school meal eligibility*								
Not eligible for free school meals	1.00	[Reference]	1.00	[Reference]	1.00	[Reference]	1.00	[Reference]
Eligible for free school meals	1.49	[1.29, 1.73]**	1.14	[0.87, 1.48]	1.68	[1.40, 2.01]***	1.22	[0.94, 1.59]
*Region*								
London	1.00	[Reference]	1.00	[Reference]	1.00	[Reference]	1.00	[Reference]
South East	1.17	[0.82, 1.68]	1.18	[0.73, 1.91]	0.90	[0.58, 1.40]	1.27	[0.63, 2.55]
East of England	0.88	[0.61, 1.27]	1.62	[0.60, 4.35]	0.59	[0.37, 0.93]	1.32	[0.50, 3.49]
West Midlands	0.96	[0.68, 1.37]	1.53	[0.79, 2.97]	0.96	[0.63, 1.48]	1.58	[0.87, 2.88]
East Midlands	2.04	[1.39, 2.99]	2.19	[1.38, 3.47]***	1.59	[0.99, 2.54]	1.57	[0.91, 2.69]
Yorkshire and the Humber	1.25	[0.87, 1.77]	2.43	[1.18, 5.03]*	0.83	[0.54, 1.29]	1.90	[0.95, 3.80]
North East	1.66	[1.20, 2.31]	2.58	[1.51, 4.42]***	1.56	[1.05, 2.33]	2.97	[1.71, 5.18]***
South West	1.31	[0.89, 1.93]	2.63	[1.09, 6.32]*	1.04	[0.65, 1.68]	2.28	[1.11, 4.66]*
Wales	1.73	[1.31, 2.29]**	2.79	[1.79, 4.36]***	1.59	[1.13, 2.23]*	2.74	[1.64, 4.57]***
North West	2.44	[1.78, 3.34]***	2.91	[1.97, 4.29]***	1.81	[1.23, 2.66]	1.91	[1.16, 3.14]**
Northern Ireland	2.48	[1.87, 3.27]***	3.63	[2.33, 5.67]***	2.76	[1.96, 3.87]***	4.42	[2.67, 7.31]***
*School type*								
Independent school	1.00	[Reference]	1.00	[Reference]	1.00	[Reference]	1.00	[Reference]
Academy or free school	2.19	[1.54, 3.11]*	1.84	[0.87, 3.90]	3.43	[2.16, 5.44]***	2.04	[1.21, 3.47]**
Secondary school	3.41	[2.45, 4.75]**	2.13	[0.99, 4.59]*	6.40	[4.13, 9.92]***	2.38	[1.36, 4.15]**
*Index of multiple deprivation quintile*								
80–100% Least deprived quintile	1.00	[Reference]	1.00	[Reference]	1.00	[Reference]	1.00	[Reference]
60–80%	1.25	[0.98, 1.61]	1.41	[0.91, 2.17]	1.19	[0.87, 1.62]	1.58	[1.02, 2.44]*
40–60%	1.33	[1.04, 1.70]	1.43	[0.87, 2.34]	1.55	[1.14, 2.09]	1.71	[1.01, 2.90]*
20–40%	1.52	[1.21, 1.92]*	1.38	[0.96, 1.99]	1.69	[1.27, 2.26]*	1.72	[1.21, 2.44]**
0–20% Most deprived quintile	1.93	[1.55, 2.39]*	1.55	[0.98, 2.44]	2.20	[1.69, 2.88]**	2.11	[1.30, 3.42]**
*Ethnicity*								
White	1.00	[Reference]	1.00	[Reference]	1.00	[Reference]	1.00	[Reference]
Non-white	0.79	[0.67, 0.93]	1.01	[0.73, 1.38]	0.70	[0.57, 0.86]	0.85	[0.60, 1.21]
*Frequency of brushing teeth*								
Twice a day or more	1.00	[Reference]	1.00	[Reference]	1.00	[Reference]	1.00	[Reference]
Once a day or less	1.36	[1.07, 1.73]**	1.26	[1.01, 1.56]*	1.40	[0.99, 1.97]*	1.20	[0.91, 1.60]
*Frequency of sugar intake*								
Less than four times a day	1.00	[Reference]	1.00	[Reference]	1.00	[Reference]	1.00	[Reference]
Four or more times a day	1.57	[1.22, 2.03]***	1.42	[1.13, 1.78]**	1.75	[1.36, 2.24]***	1.54	[1.20, 1.99]***
*Usual dental attendance*^*c*^								
Regular	1.00	[Reference]	1.00	[Reference]	1.00	[Reference]	1.00	[Reference]
Irregular/none	1.82	[1.54, 2.15]***	2.18	[1.58, 3.00]***	2.05	[1.67, 2.52]***	2.75	[1.95, 3.87]***
*Self-rated dental anxiety score MDAS grouping*								
Low/no anxiety (score 5–9)	1.00	[Reference]	1.00	[Reference]	1.00	[Reference]	1.00	[Reference]
Moderate anxiety (score 10–18)	0.93	[0.82, 1.07]***	0.73	[0.60, 0.89]**	0.87	[0.74, 1.02]***	0.68	[0.53, 0.87]**
Extreme anxiety (score 19–25)	1.28	[1.05, 1.57]	0.99	[0.76, 1.30]	1.39	[1.09, 1.79]	1.06	[0.77, 1.46]

^a^D_AV_MFS (CDHS AV and above) represents decay experience *according to clinical decay* threshold. D_2V_MFS (CDHS 2 V and above) represents decay experience *obvious decay* threshold

^b^Unadjusted and full-adjusted Negative binomial regression models were fitted, rate ratios (RR) were reported

^c^Regular represents “for a check-up”, irregular/none combines “only when have trouble with teeth” and “never been to the dentist”

*p < 0.05, **p < 0.01, ***p < 0.001

After adjusting for sociodemographic factors in 12-year-olds, geographic factors relating to the country/region of England, area deprivation, free school meals, white ethnicity, remained significant, together with behavioural factors relating to sugar and dental attendance. Higher frequency sugar consumption (four or more times per day), and irregular/no dental attendance, emerged as the leading behavioural risk factors for *clinical decay* at tooth surface level. In relation to *obvious decay* amongst 12-year-olds, similar patterns were present, the only differences being that school type was also significant, whilst high frequency sugar consumption was not (Table 4).

After adjusting for personal and socio-economic factors in 15-year-olds, social factors relating to the country/region of England, gender, school type, behavioural factors including higher frequency sugar consumption, less frequent toothbrushing and irregular/no dental attendance emerged as risk factors for *clinical decay* at tooth surface level*,* whereas reporting moderate anxiety to be a protective factor. In relation to *obvious decay*, similar patterns were present, the only differences being that area deprivation was statistically significant and toothbrushing was not (Table 5).

## Discussion

### Summary of findings

This study provides important insights into the pattern of dental caries at tooth surface, tooth, and individual level in a high-income country where dental caries, despite a recent decline, remains the most prevalent condition in childhood. Visualisation of the distribution of dental caries at different stages of carious process across every surface of permanent dentition in 12- and 15-year-old children from England, Wales, and Northern Ireland shows the burden of disease carried by each tooth, most notably first permanent molars. Examination of two diagnostic thresholds: *clinical decay* which includes enamel caries and represents the criteria used by clinicians examining and providing care; and *obvious decay* which relates to previous epidemiological survey thresholds and is consistent with the WHO oral health surveys basic methods [26], highlights the volume of initial caries lesions (enamel caries) in these children, particularly 12-year-olds. The relative merits of using both of these thresholds have been debated in Europe and interested organisations have produced a recent “Brussels Statement” setting out the needs of modern caries epidemiology in Europe and beyond [27]. It is clear from these data that using the *clinical decay* threshold provides a more complete and higher representation of disease at population level, with implications for both clinical care and health policy. The findings also highlight the importance of social and behavioural factors, together with regional variation.

### Epidemiology implications

It is very clear from these findings that the threshold of reporting dental caries in epidemiological studies requires careful consideration, as surveys which just focus on *obvious decay* seriously under-report the prevalence of disease, which increases with age cohort. If epidemiological surveys focus on code CDHS 2V and above (equivalent to ICDAS 4 and above) as the diagnostic threshold for decay [17], they will miss at least 40% of the dental caries. Caries lesions vary in their likelihood of transition in that they may progress, arrest or regress [28]. Although national surveys do not typically assess the activity status of lesions, there is evidence from Guedes et al. and Ferreira Zandoná et al. that around 10–11.9% of the active non-cavitated enamel lesions and 46–50% of cavitated enamel surfaces progress to frank cavitation within a 2-year period [9, 29]. This will of course depend on any management, or not, of individual risk factors. Our cross-sectional data on adolescents do however highlight the higher levels of both *clinical* and *obvious decay* in 15-year-olds compared with 12-year-olds, drawn from the same contexts, suggesting that there is greater potential for risk management given that enamel lesions are potentially reversible [13]. Therefore, data on the volume of enamel caries provide an indication of great preventive opportunity [30] and should be taken into account by clinicians.

### Clinical implications

If readers consider that most lesions in 12-year-olds were in enamel, this suggests that progression of much future disease could be prevented by supporting young people to lower free sugar consumption to ensure it doesn’t exceed 5% of dietary energy in line with contemporary evidence [31], increase toothbrushing with a fluoride toothpaste, and apply fluoride varnish regularly [32]. This presents a large opportunity for prevention early in adolescence. Implementing preventive guidance such as Delivering Better Oral Health [32], can support these children effectively through managing risk. Examples include behavioural changes in swopping sugar sweetened beverages and snacks for healthy snacks, as outlined in the *Change 4 Life* programme, particularly between meals, is important [33]. If we do not do this, we fail the children themselves, and the healthcare system, leading to higher disease treatment and retreatment, with its associated costs for individuals and government.

### Children: patterns within the oral cavity

Establishing health patterns of behaviour in support of oral health is an important foundation of maintaining a ‘functional dentition’ through adulthood [34]. The proportions of dental caries experience that involved enamel decay in all surfaces in 15-year-olds was around 10% lower than in 12-year-olds, whilst more lesions were treated or had *obvious decay*. Furthermore, the prevalence, level and intensity of decay increased by age with more surfaces, teeth and children affected in this cross-sectional survey. As expected, occlusal and buccal surfaces of first molars as well as lower second molars were most effected by dental caries and either restored or in need of dental intervention. This suggests that the surface-/tooth-level of caries prevalence exhibited overall symmetry. Horizontal (left/right) symmetry, which indicated similar propensity of decay affected for both sides of the same dentition can be observed in most tooth sites, whilst a degree of vertical symmetry (upper/lower) was present in relation to disease in posterior sextants. Whilst it has traditionally been considered that mandibular molars are more vulnerable to dental caries comparing with their maxillary counterparts as indicated by Luan et al. [35] who conducted a ten year follow-up study among the Chinese population, our results find support in agreement with Macek’s work in the United States [36]. We found that first molars which have been present in the oral cavity for over 6 years show little difference in *clinical decay* experience vertically or horizontally. Similarly, Macek et al. [36], reported that maxillary and mandibular first molars among 19.5-year-olds in U.S., shared similar relative susceptibility to dental caries. Batchelor and Sheiham [37] also suggested that occlusal fissured surfaces of the first molar teeth, and buccal pits sites on lower first molars could be grouped together according to their similar caries susceptibility.

Interestingly, symmetry of caries prevalence doesn’t illustrate these teeth/tooth surfaces necessarily suffered from same stages of caries simultaneously in a certain child, which hasn’t been emphasized before. The proportion of pupils with decayed lower second molars who’s upper second molars suffered from caries at the same time, increased from 47% in 12-years to 68% in 15-years with age cohort. It is quite possible that a tooth/surface is in a very early (subclinical) stage of caries which cannot be detected by clinical visual examination, meanwhile, its asymmetrical surface already progressed to more advanced signs (white spot or cavitation). The similar pattern of caries horizontally and to an extent vertically in the same type of teeth suggests that when an effect that reduces/increases the cariogenic process of one of the teeth in children, is likely to affect their counterparts in the other three quadrants. This was supported by Batchelor and Sheiham’s findings that occlusal surfaces of second molars and buccal sites on mandibular second molars were in the same group in order of caries susceptibility when 5- to 16-year-olds were involved [37]. Differences vertically seem to reduce with increasing length of time in the oral cavity.

### What do the models tell us that will support clinical care and community action?

Modelling these data suggests that dental caries prevalence at surface level for *clinical/obvious decay* thresholds was associated with similar but not identical dental behavioural and psychological factors in two age groups, albeit that not always significant. Sugar intake frequency, one of the most recognised dental caries risk factors, was shown to be related to *clinical/obvious decay* experience (D_AV/2V_MFS) in this research, which is consistent with the finding of 4^th^ National Oral Health Survey in China [2], and the body of evidence reported by Moynihan and colleagues in their important systematic review [38]. The WHO strongly recommends a reduced intake of free sugars (include monosaccharides and disaccharides added to foods and beverages by the manufacturer, cook or consumer, and sugars naturally present in honey, syrups, fruit juices and fruit juice concentrates) to less than 10% of total energy intake throughout the life-course [6], affirmed within the UK at the 5% level [31, 39, 40]. Toothbrushing plays an important role in delivering fluoride toothpaste and should be advised in line with current evidence [32]. Whilst attending a dentist does not necessarily prevent disease it increasingly can assist with disease prevention in young people through delivery of fluoride varnish and fissure sealants, together with advice on fluoride and diet [19, 32]. There remains a lack of consensus on the relationship between dental anxiety and dental caries [41–43]. Dental anxiety was reported to predict caries incidence in 15- to 18-year olds [42], but was not significantly associated with dental caries experience at age 12–15 years [41]. Interestingly, between the different age-groups examined (12- and 15-year-olds), moderate dental anxiety was verified as a strongly protective factor in this study for the first time. This may possibly be explained by the fact that these children have had a heightened awareness of dentistry and other input such as orthodontic treatment (with possible extractions due to crowding) and greater preventive input; however, this should be investigated further. Overall, these findings suggest the importance of evidence-based preventive care supported by regular dental attendance, particularly if dentists are practicing preventive and minimally invasive dentistry.

Variation in the significance of the findings of the analysis could relate to the year cohort, sampling or the instruments utilized for data collection. What is clear is that the same patterns are present across both ages. Importantly the patterns and trends were similar in relation to significant or tending towards significance in both adjusted models.

The limitations of the study include the fact that it used cross-sectional data and differences between study samples and excluded samples could be found which have implications for its representativeness. None-the-less the uptake of the self-complete questionnaire survey was high and the survey was innovative in providing the opportunity to compare the data retrospectively with past surveys to document a further decline in caries [30], as well as being epidemiologically innovative. Ideally, it would be good to have longitudinal data to provide a better representation of caries trajectories, as with the Dunedin study [44].

The present findings have very important implications for public health policy, starting with epidemiology. First, initial stage of dental caries occupied almost half of decay-experienced surfaces/teeth in children according to information of CDHS 2013. Thus, if epidemiological surveys merely focus on *obvious decay* to achieve comparability with past surveys, the findings will seriously underestimate the prevalence of disease, and provide limited insight to the planning of health interventions. Reporting dental caries levels at the *clinical decay* threshold is increasingly important and possible, and the methodology used by the CDHS 2013 survey can be useful to other countries and should be replicated in the UK in future surveys. Second, the volume of enamel caries in both age-groups, highlights the important opportunity to recognise and arrest progression of these non-cavitation lesions—if they are controlled, many restorations and repeated restorations will be prevented with potential cost savings [27, 30]. Action is required by all parties to alter children’s risk of oral disease. Third, caries susceptibility follows a clear pattern with horizontal symmetry, and certain vertical symmetry at patient-level with these modest differences reducing with age. This tendency indicates high requirements of early dental interventions by clinicians, i.e. pit and fissure sealing and preventive resin restoration, particularly in first molars and second molars whose asymmetrical teeth already infected by dental decay. Fourth, and finally, given the pattern of disease in society and multiple risk factors, further research needs to be addressed to explore a way of categorising individuals into different dental caries affected patterns.

## Conclusion

In conclusion this research highlights the importance of recognising the dental caries patterns in epidemiological surveys, and the importance of appreciating the caries process and being clear about dental caries thresholds in the population. Secondary analysis illustrates that working with and examining different caries thresholds, including initial stage lesions, can provide better insights into dental caries staging and prevalence to inform contemporary primary care, which includes supporting self-care.

## Supplementary Information

**Additional file 1**. **Appendix Figure 1**. Distribution of CDHS 2013 code in each surface of 12-year-olds in England, Wales, and Northern Ireland, 2013 (n = 2532). **Appendix Figure 2**. Distribution of CDHS 2013 code in each surface of 15-year-olds in England, Wales, and Northern Ireland, 2013 (n = 2160).

## Data Availability

The dataset supporting the conclusions of this article is available in the public UK data service website, available from: http://doi.org/10.5255/UKDA-SN-7774-1.

## Abbreviations

CDHS 2013: 2013 Children’s Dental Health Survey
ICDAS: International Caries Detection and Assessment System
UK: United Kingdom
RRs: Rate ratios
CI: Confidence intervals
