# Supplementary material for: Dental caries thresholds among adolescents in England, Wales, and Northern Ireland, 2013 at 12, and 15 years: implications for epidemiology and clinical care
Source: BMC Oral Health. 2021 Mar 19;21:137. doi: 10.1186/s12903-021-01507-1 (PMC7980596; doi:10.1186/s12903-021-01507-1)
Supplement: Supplementary file 1 — Additional file 1. Appendix Figure 1. Distribution of CDHS 2013 code in each surface of 12-year-olds in England, Wales, and Northern Ireland, 2013 (n = 2532). Appendix Figure 2. Distribution of CDHS 2013 code in each surface of 15-year-olds in England, Wales, and Northern Ireland, 2013 (n = 2160). [file 12903_2021_1507_MOESM1_ESM.docx]

**Supplementary Material**

**Dental Caries Thresholds among Adolescents in England, Wales and Northern Ireland, 2013 at 12, and 15 years: Implications for Epidemiology and Clinical Care**

**Xiaozhe Wang,**^1^ **Eduardo Bernabe,^2^ Nigel Pitts,^3^ Shuguo Zheng,**^1^ **Jennifer E Gallagher ^4^**

^1^ Department of Preventive Dentistry, Peking University School and Hospital of Stomatology & National Clinical Research Center for Oral Diseases & National Engineering Laboratory for Digital and Material Technology of Stomatology & Beijing Key Laboratory of Digital Stomatology, Beijing, PR China

2 King’s College London, Centre for Host Microbiome Interactions, Denmark Hill Campus, Bessemer Road, London, SE5 9RS, UK

3 King’s College London, Centre for Clinical and Translational Research, Guy’s Hospital Campus, Great Maze Pond, London, SE1 9RT, UK

4 King’s College London, Faculty of Dentistry, Oral & Craniofacial Sciences, Bessemer Road, London, SE5 9RS, UK

**Corresponding author(s)**

*Correspondence to:

**Prof Jennifer E Gallagher MBE**

**Dean for International Affairs**

**Newland-Pedley Professor of Oral Health Strategy/Hon Consultant in Dental Public Health**

**King’s College London, Faculty of Dentistry, Oral & Craniofacial Sciences, Bessemer Road, London, SE5 9RS, UK**

**Telephone 02032995171/3481 Admin (research)**

**Email:** [**jenny.gallagher@kcl.ac.uk**](mailto:jenny.gallagher@kcl.ac.uk)

**Dr Xiaozhe Wang**

**Attending Dentist**

**Department of Preventive Dentistry, Peking University School and Hospital of Stomatology & National Clinical Research Center for Oral Diseases & National Engineering Laboratory for Digital and Material Technology of Stomatology & Beijing Key Laboratory of Digital Stomatology, 22 Zhongguancun Avenue South, Haidian District, Beijing 100081, PR China**

**Telephone 08601082195558**

**Email: neptunewxz@163.com**


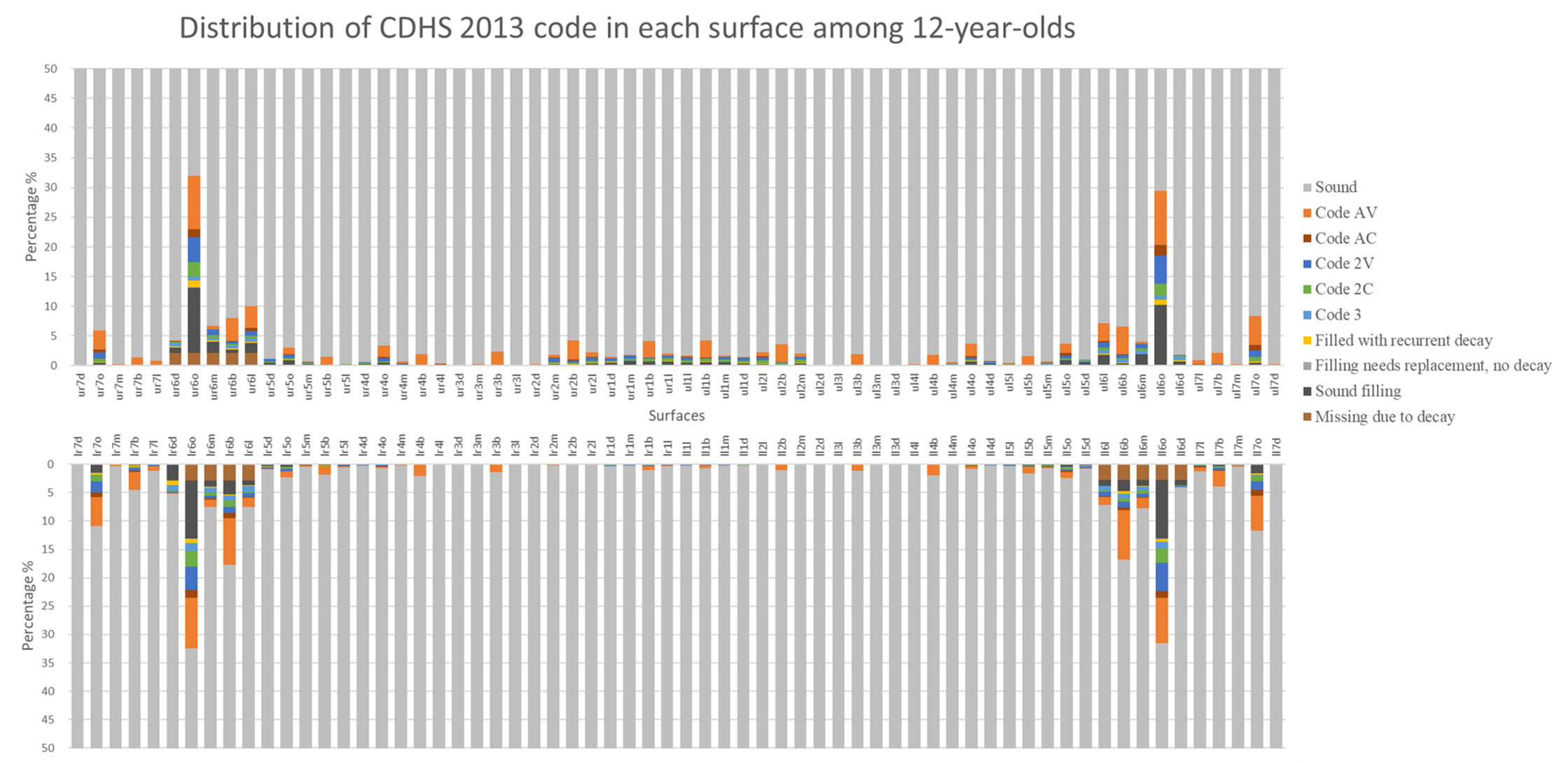


**Appendix Figure 1. Distribution of CDHS 2013 code in each surface of 12-year-olds (n = 2,532)**


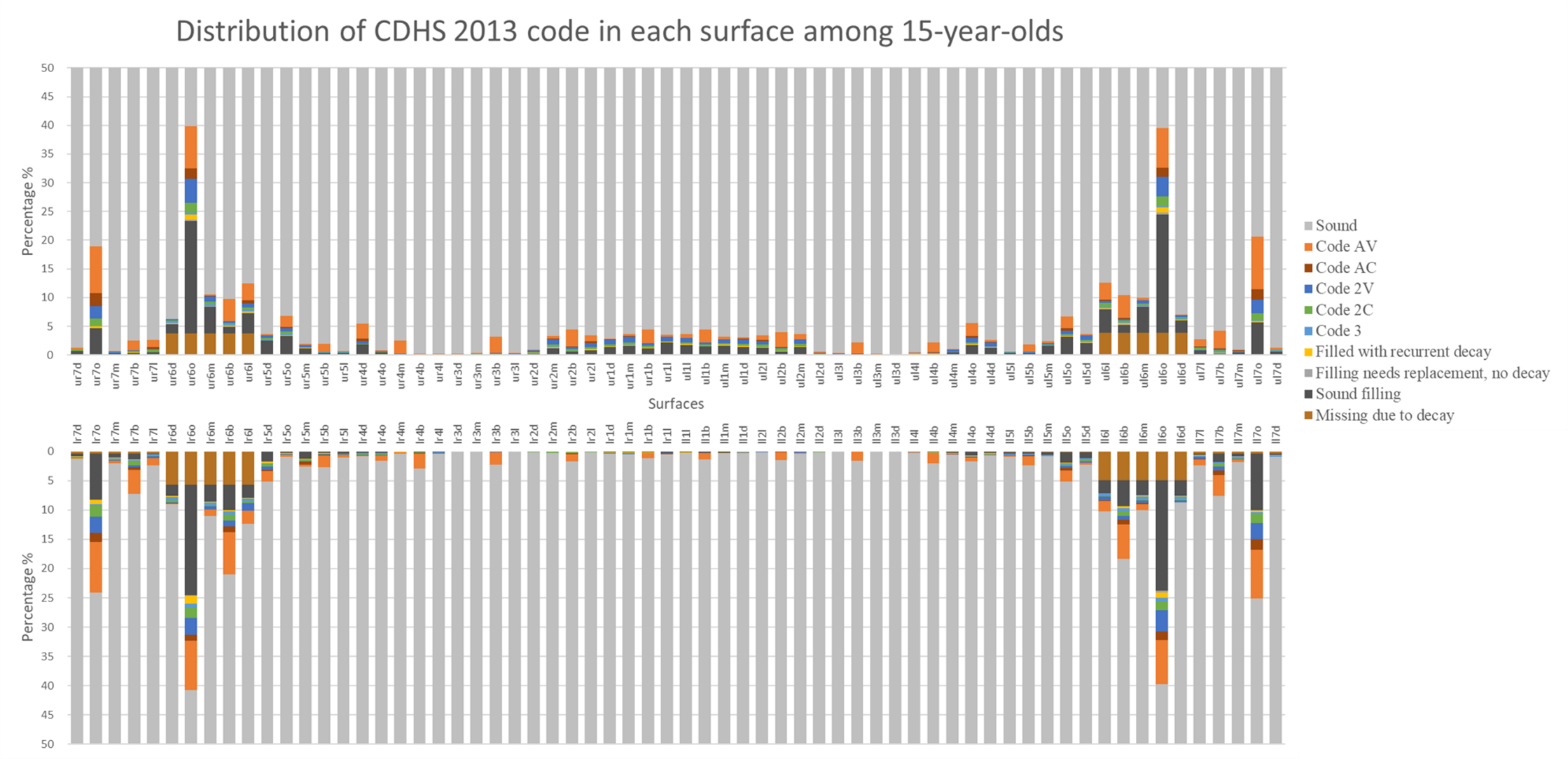


**Appendix Figure 2. Distribution of CDHS 2013 code in each surface of 15-year-olds (n = 2,418)**
